# Perceived level of knowledge and skills about clinical management of adolescent idiopathic scoliosis among undergraduate chiropractic students and clinical faculty at a Canadian chiropractic program: cross–sectional study

**DOI:** 10.1186/1748-7161-9-S1-O60

**Published:** 2014-12-04

**Authors:** Chantal Doucet, André Bussières, Catherine Fradette

**Affiliations:** 1 University Trois-Rivières, Québec, Canada; 2 McGill University, Montreal, Canada

## Background

The prevalence of adolescent idiopathic scoliosis (AIS) ranges from 0.93 to 5.2%. Among these individuals ,27% will consult primary care providers for back pain, including chiropractors. Undergraduate chiropractic curriculum include training on the management of AIS. Recent studies however suggest such training may be sub-optimal to ensure sufficient clinical competency. To address this, a four session training educational workshop was provided to a group of chiropractic interns and clinical faculty members at l’Université du Québec à Trois-Rivières (UQTR).

## Aim

To determine the current level of knowledge and skills about AIS management among junior, senior interns and the clinical faculty members at the Outpatient clinic at UQTR.

## Design and methods

A cross-sectional online survey was administered to 112 interns and clinical faculty members at UQTR outpatient clinic to assess self-reported levels of knowledge and skills about AIS. The survey questionnaire was pilot tested prior to distribution. Three groups of respondents (juniors, seniors interns and clinicians) completed 15 closed-ended questions on AIS clinical presentation, risks factors and management. Responses were compared between groups using the Fisher’s exact test.

## Results

A response rate of 43% (n=48/112) was obtained from 14 juniors and 19 seniors interns and 15 clinical faculty members. Among the three groups, 93% of clinicians considered having moderate to high level of knowledge on AIS, compared with 73% senior interns and 21% junior interns (p=0.0001). The proportion of interns and clinicians exposed to an educational training intervention significantly differed between groups ( p≤0.0201) with fewer juniors interns 57% and clinicians 73% attending than seniors interns 95%. The level of awareness on the existence of practice guidelines on the management of AIS varied similarly across groups (p≤0.0138), with less awareness among junior interns 29% and clinicians 50% than senior interns 79%.

## Conclusion

Study results suggest a persistent knowledge gap among interns and clinical faculty members for the management of AIS in a chiropractic teaching institution. Guideline dissemination and implementation strategies are needed to fulfill these gaps to improve patient care in this setting.

